# Impact of aldosterone-producing adenoma on cardiac structures in echocardiography

**DOI:** 10.1007/s12574-013-0168-y

**Published:** 2013-02-05

**Authors:** Takayuki Hidaka, Tsuguka Shiwa, Yuichi Fujii, Kenji Nishioka, Hiroto Utsunomiya, Ken Ishibashi, Naoya Mitsuba, Yoshihiro Dohi, Noboru Oda, Kensuke Noma, Satoru Kurisu, Yukiko Nakano, Hideya Yamamoto, Takafumi Iishida, Yukihito Higashi, Yasuki Kihara

**Affiliations:** 1 Department of Cardiovascular Medicine, Hiroshima University Graduate School of Biomedical Sciences and Health, Kasumi 1-2-3, Minami-ku, Hiroshima Japan; 2 Department of Molecular and Internal Medicine Endocrinology and Diabetic Medicine, Hiroshima University Graduate School of Biomedical Sciences and Health, Kasumi 1-2-3, Minami-ku, Hiroshima Japan; 3 Department of Regeneration and Medicine Research Center for Radiation Genome Medicine, Research Institute for Radiation Biology and Medicine, Hiroshima University, Kasumi 1-2-3, Minami-ku, Hiroshima Japan

**Keywords:** Echocardiography, Aldosterone, Hypertrophy, Hypertension

## Abstract

**Background:**

Primary aldosteronism (PA) is a most common cause of secondary hypertension. In PA, left ventricular hypertrophy (LVH) is more progressive than in any other cause of hypertension. Aldosterone-producing adenoma (APA) and idiopathic hyperaldosteronism (IHA) are major subtypes of PA. However there is little information concerned with differences of cardiac structures between these two subtypes.

**Methods:**

We reviewed echocardiographic findings in 46 patients with PA. All patients had a positive screen test and subtypes of PA were confirmed by adrenal vein sampling. Subjects consisted of 20 patients with APA (APA group, 52.4 ± 10.8 years) and 26 patients with IHA (IHA group, 56.2 ± 9.5 years). We investigated differences of cardiac structures and functions in the left atrium and ventricle between the APA group and IHA group.

**Results:**

In terms of clinical characteristics, the height and duration of hypertension were greater and serum potassium concentration and BMI were lower in the APA group than in the IHA group. Plasma aldosterone concentration (PAC) and PAC to plasma renin activity ratio were higher in the APA group than in the IHA group. In echocardiographic assessment, the left atrial volume, left ventricular end-diastolic and end-systolic diameters, left ventricular mass (LVM), and prevalence of LVH were greater in the APA group than in the IHA group. Multiple linear regression analysis revealed that the diagnosis of APA independently correlated with left atrial volume, left ventricular end-diastolic diameter, and LVM.

**Conclusions:**

We demonstrated that differences of cardiac structures between the APA group and IHA group existed. In APA, left atrial enlargement and LVH were more prominent than in IHA.

## Introduction

Primary aldosteronism (PA) is a common cause of secondary hypertension. Aldosterone-producing adenoma (APA) and idiopathic hyperaldosteronism (IHA) are major subtypes of PA [1]. Distinction between APA and IHA is essential because of differential therapeutic strategies. The widespread use of the plasma aldosterone to renin ratio (ARR) as a screening test leads to the increase in the number of patient who need definitive tests such as adrenal vein sampling (AVS) to determine the therapeutic strategy [2]. However, AVS is invasive and technically difficult and requires considerable expertise. To distinguish between APA and IHA, it would be necessary to establish non-invasive and simple methods.

Left ventricular hypertrophy (LVH) is one of the most important predictors of cardiovascular events. Previous studies reported that, in patients with PA, LVH preceded other organ damage [3] and was more exaggerated than in the patients with other causes of secondary hypertension [4]. Some experimental studies have shown that excess aldosterone promotes extracellular matrix and collagen deposition leading to LVH and impairment of left ventricular diastolic properties [5–10]. Excess aldosterone also affects left ventricular morphology through an increase in sodium and water retention. Some studies reported that the relationship between cardiac structure and plasma aldosterone concentration (PAC) is affected by several factors such as existence of heart failure, race, sex, obesity, and sodium intake [11–13]. However, there has been little information about the difference of left ventricular structures and function among subtypes of PA such as APA and IHA. The purpose of this study was to investigate the morphological and functional difference of the left atrium and ventricle between patients with APA and IHA.

## Methods

### Subjects

We investigated 46 consecutive hypertensive patients with a positive screening test for PA who were referred to our department for AVS from February 2009 to January 2012. All these patients were suspected of having secondary hypertension. Echocardiographic assessment was performed in all patients on their first visit. Blood pressure (BP) and heart rate (HR) were measured at the time of echocardiographic examination without the cessation of antihypertensive agents. The hormonal evaluation was conducted within 2 months after the echocardiographic assessment. No patient had a history of ischemic heart disease, valvular heart disease, or any other cardiomyopathies. Subjects consisted of 20 patients with APA (APA group) and 26 patients with IHA (IHA group).

### Diagnosis of primary aldosteronism

The aldosterone receptor blockers, angiotensin receptor blockers, and β-blockers were withdrawn and changed to long-acting calcium channel blockers, if needed, at least 2 weeks before the hormonal evaluation. Administration of long-acting calcium channel blockers had been continued. The PAC (in ng/dl) and the plasma renin activity (PRA) (in ng/ml/h) were measured after overnight fast and 30 min rest. A positive screening test for PA was defined as a PAC value greater than 15 ng/day and a PAC/PRA ratio (ARR) greater than 20 in accordance with the Japanese Society of Hypertension’s guideline for the management of hypertension 2009. The AVS protocol and diagnostic criteria were in accordance with the guideline of the Japan Endocrine Society [2]. These measurements were conducted within 2 months after echocardiographic assessment.

### Echocardiography

Comprehensive echocardiographic assessment was conducted by three experienced sonographers, who had no knowledge of the clinical data, using high-quality commercially available ultrasound systems (GE Healthcare Vivid 7, Milwaukee, WI; TOSHIBA Medical Artida, Tokyo). All echocardiographic parameters were measured according to the recommendation of the American Society of Echocardiography [14, 15]. Images were obtained in the parasternal (long and short axis) and apical views. Left atrial volume, left ventricular volume, and ejection fraction were calculated from apical two- and four-chamber views using the method of discs. The left ventricular mass (LVM) was calculated by the Devereux method [16, 17] and indexed by body surface area [left ventricular mass index (LVMI)] and LVH was defined as LVMI greater than 115 g/m^2^ in men and greater than 95 g/m^2^ in women. Other echocardiographic parameters were normalized for body surface area if needed. LV diameters and LV posterior wall thickness (LVPWT) and interventricular septal thickness (IVST) were measured at the level of the tip of the mitral valve leaflet using B-mode images. Relative wall thickness (RWT) was calculated according to the following equation: RWT = (IVST + LVPWT)/left ventricular end-diastolic diameter.

Stroke volume was determined as the product of the time–velocity integral measured at the left ventricular outflow tract and the cross-sectional area of the aortic annulus.

Transmitral flow velocity was measured using pulsed wave Doppler from the apical long axis view at the end of expiration. The sample volume was positioned between the tips of the mitral leaflet maintaining the ultrasonic beam parallel to the direction of blood flow. The following parameters were obtained: early diastolic peak flow velocity (*E*), peak flow velocity at atrial contraction (*A*), *E*/*A* ratio, and deceleration time of *E* (Dct). Mitral annular velocity was measured using pulsed wave tissue Doppler imaging (TDI). Early diastolic peak velocity (*e*′), late diastolic velocity (*a*′), and systolic velocity (*s*′) were measured at the septal and lateral sides of the mitral annulus from the apical four-chamber view. Systolic pulmonary artery pressure (SPAP) was estimated using continuous wave Doppler by calculating the right ventricular to right atrial pressure gradient during systole, approximated by the modified Bernoulli equation as 4*v* ^2^, where *v* is the tricuspid regurgitation velocity. Right atrial pressure was estimated on the basis of echocardiographic assessment of the inferior vena cava and added to the calculated gradient to give the SPAP.

### Statistical analysis

Data are expressed as mean ± SD or median (interquartile range) as indicated and the comparison between groups was performed with Student’s *t* test for unpaired data or the Mann–Whitney test as appropriate. Categorical variables were compared by the Chi-squared test using Fisher’s exact method. Results were considered significant at *p* < 0.05. The relationship between variables was investigated using univariate and multiple linear regression analysis. The data were processed using the software package Stat View V (SAS Institute Inc., Cary, North Carolina, USA).

## Results

### Clinical characteristics

Clinical characteristics in the APA group and IHA group are summarized in Table 1. In the APA group, the duration of hypertension was longer and BMI and serum potassium concentration were lower than in the IHA group. BP and HR measured at the time of echocardiographic examination and serum creatinine concentration were similar between the two groups. The number of antihypertensive agents used per patient and the duration of hypertension were greater in the APA group than in the IHA group. No patient was treated with angiotensin-converting enzyme inhibitors and diuretics. Hormonal data are shown in Table 2. PAC and ARR were significantly higher in the APA group compared with the IHA group. PRA was similar between the two groups.

**Table 1:** Clinical characteristics in patients with APA and IHA

Variables	APA (*n* = 20)	IHA (*n* = 26)	*p* value
Age, years	52.4 ± 10.8	56.2 ± 9.5	NS
Female sex, *n* (%)	7 (35)	15 (58)	NS
Height, m	1.66 ± 0.69	1.60 ± 0.78	0.006
Weight, kg	67.7 ± 14.5	62.3 ± 11.6	NS
Body mass index, kg/m^2^	21.9 ± 1.2	23.0 ± 1.3	0.009
Blood pressure, mm Hg			
Systolic	145.6 ± 20.6	139.0 ± 17.7	NS
Diastolic	85.3 ± 15.2	83.9 ± 13.0	NS
Heart rate, bpm	67.8 ± 7.6	69.3 ± 10.3	NS
Hemoglobin, g/dl	13.5 ± 1.8	14.0 ± 1.9	NS
Creatinine, mg/dl	0.83 ± 0.24	0.73 ± 0.15	NS
Na, mEq/l	142.3 ± 3.2	140.9 ± 2.8	NS
K, mEq/l	3.5 ± 0.5	4.1 ± 0.4	0.0001
Cl, mEq/l	102.6 ± 4.0	104.3 ± 20.4	NS
Duration of hypertension, years	11.6 ± 0.9	7.4 ± 9.4	0.04
Medications, *n* (%)			
ARB	2	1	NS
β-Blocker	4	2	NS
CCB	17	16	NS
Number of antihypertensive agents, *n*	1.2 ± 0.8	0.7 ± 0.7	0.03

Body mass index (BMI) (kg/m^2^) = weight (kg)/height (m)^2^. The body surface area (BSA) (m^2^ BSA) = 88.83 × height (cm)^0.663^ × weight (kg)^0.444^

*ARB* angiotensin II receptor blocker, *CCB* calcium channel blocker,* NS* not significant

**Table 2:** Hormonal data in patients with APA and IHA

Variables	APA (*n* = 20)	IHA (*n* = 26)	*p* value
PAC (ng/dl)			
Median	37.6	13.3	<0.0001
Interquartile range	20.3–76.3	10.4–16.1	
PRA (ng/ml/h)			
Median	0.30	0.30	NS
Interquartile range	0.15–0.40	0.2–0.4	
ARR			
Median	132.7	39.8	<0.0005
Interquartile range	52.4–472.7	26.3–62.0	

*PAC* plasma aldosterone concentration, *PRA* plasma renin activity, *ARR* the ratio of plasma aldosterone to plasma renin activity,* NS* not significant

### Echocardiographic findings

Echocardiographic parameters in the APA group and IHA group are summarized in Table 3. Left atrial volume, left ventricular end-diastolic diameter and end-systolic diameter, stroke volume, and diameter of the inferior vena cava were larger in the APA group than in the IHA group. Left ventricular ejection fraction was similar between the two groups. Left ventricular wall was thicker in the APA group than in the IHA group. The RWT was similar between the two groups. LVMI was larger in the APA group than in the IHA group. The prevalence of LVH was significantly higher in APA than in IHA. Transmitral flow velocities, except for *E*, were similar between the two groups. *E* was higher in the APA group than in the IHA group. *E*/*A* ratio was similar between the two groups. Mitral annular velocities and *E*/*e*′ were similar between the two groups. Tricuspid regurgitation velocity and SPAP were similar between the two groups.

**Table 3:** Echocardiographic data in patients with APA and IHA

Variables	APA (*n* = 20)	IHA (*n* = 25)	*p* value
Left atrium			
Diameter, cm/m^2^ BSA	2.2 ± 0.2	2.1 ± 0.2	NS
Volume, ml/m^2^ BSA	40.4 ± 14.1	29.9 ± 7.6	0.002
Left ventricle			
End-diastolic diameter, cm/m^2^ BSA	3.0 ± 0.2	2.8 ± 0.3	0.014
End-systolic diameter, cm/m^2^ BSA	1.9 ± 0.2	1.8 ± 0.2	0.0021
Interventricular septal wall thickness, cm	1.0 ± 0.2	0.9 ± 0.1	0.003
Posterior wall thickness, cm	1.1 ± 0.2	0.9 ± 0.1	0.013
Relative wall thickness	0.42 ± 0.08	0.39 ± 0.07	NS
Ejection fraction, %	64.3 ± 4.6	64.3 ± 2.7	NS
Mass, g/m^2^ BSA	150.4 ± 30.6	110.8 ± 15.9	0.0001
Stroke volume, ml/m^2^ BSA	56.6 ± 10.9	43.6 ± 12.7	0.025
Prevalence of left ventricular hypertrophy, *n* (%)	20 (100)	17 (68)	0.006
Transmitral flow			
*E*, cm/s	77.5 ± 25.3	65.6 ± 13.3	0.046
*A*, cm/s	66.2 ± 14.1	69.6 ± 13.0	NS
*E*/*A*	1.1 ± 0.3	1.0 ± 0.3	NS
Dct, ms	218.8 ± 42.7	220.5 ± 54.4	NS
Mitral annular velocity			
Septal			
*e*′, cm/s	8.0 ± 2.4	6.8 ± 1.8	NS
*a*′, cm/s	9.5 ± 2.3	9.9 ± 2.3	NS
*s*′, cm/s	7.8 ± 1.5	8.0 ± 1.6	NS
*E*/*e*′	9.7 ± 2.4	9.9 ± 2.7	NS
Lateral			
*e*′, cm/s	10.1 ± 2.9	9.6 ± 2.9	NS
*a*′, cm/s	9.7 ± 2.6	10.8 ± 2.6	NS
*s*′, cm/s	8.4 ± 1.8	9.1 ± 1.8	NS
*E*/*e*′	7.8 ± 2.1	7.1 ± 2.0	NS
Tricuspid regurgitation velocity, m/s	2.2 ± 0.4	2.2 ± 0.4	NS
Diameter of Inferior vena cava, cm	1.6 ± 0.4	1.3 ± 0.3	0.01
SPAP, mmHg	27.4 ± 7.5	24.7 ± 7.2	NS

Values are mean ± SD

*E* early diastolic peak flow velocity, *A* peak flow velocity at atrial contraction, *Dct* deceleration time of *E*, *e*′ mitral annular early diastolic peak velocity, *a*′ mitral annular peak velocity at atrial contraction, *s*′ mitral annular systolic peak velocity, *SPAP* systolic pulmonary pressure

### Relationship between clinical characteristics and cardiac structure

In univariate analysis, the duration of hypertension, diagnosis of APA, and log PAC significantly correlated with left atria volume index (LAVI), left ventricular end-diastolic diameter index (LVDdI), and LVMI (Table 4). Systolic blood pressure (sBP) measured at the time of echocardiographic examination significantly correlated with LVMI and tended to correlate with LAVI and LVDdI (Table 4). Multiple linear regression analysis was performed to assess the independent effects of clinical factors on cardiac structures. These factors included age, sBP, BMI, duration of hypertension, diagnosis of APA, and log PAC. Because an intercorrelation was observed between diagnosis of APA and log PAC (*r* = 0.66, *p* < 0.01), two independent models were tested. The diagnosis of APA was included in model 1 and log PAC was included in model 2. Multiple linear regression analysis revealed that the duration of hypertension independently correlated with LAVI in both models and LVDdI in model 1 (Table 5). The diagnosis of APA independently correlated with LAVI, LVDdI, and LVMI in model 1 and log PAC independently correlated with LAVI and LVMI in model 2.

**Table 4:** Univariate analysis between echocardiographic findings and clinical characteristics

Variables	LAVI		LVDdI		LVMI	
	*R*	*p* value	*R*	*p* value	*R*	*p* value
Age	−0.14	0.35	−0.26	0.08	0.02	0.34
Female sex	−0.03	0.82	−0.32	0.028	−0.27	0.07
sBP	0.26	0.08	0.27	0.07	0.36	0.016
BMI	0.047	0.76	0.063	0.09	0.27	0.08
Duration of hypertension	0.36	0.015	0.43	0.003	0.31	0.038
Diagnosis of APA	0.19	0.002	0.27	0.002	0.65	<0.0001
Log PAC	0.31	0.034	0.31	0.037	0.43	0.0033

Left atrial volume index (LAVI) (ml/m^2^) = left atrial volume (ml)/BSA (m^2^), left ventricular end-diastolic diameter index (LVDdI) = left ventricular end-diastolic diameter (cm)/BSA (m^2^), left ventricular mass index (LVMI) = left ventricular mass (g)/BSA (m^2^)

**Table 5:** Multiple linear regression analysis between echocardiographic findings and clinical characteristics

Variables	LAVI				LVDdI				LVMI			
	Model 1		Model 2		Model 1		Model 2		Model 1		Model 2	
	*R*	*p* value	*R*	*p* value	*R*	*p* value	*R*	*p* value	*R*	*p* value	*R*	*p* value
Age	−0.05	0.72	−0.07	0.37	−0.14	0.29	−0.06	0.68	0.01	0.94	0.04	0.77
Female sex	0.05	0.76	0.01	0.58	−0.19	0.24	0.15	0.44	−0.10	0.51	0.07	0.72
sBP	0.14	0.32	0.14	0.34	0.07	0.57	0.08	0.62	0.20	0.11	0.23	0.11
BMI	0.30	0.08	0.21	0.23	0.12	0.47	0.22	0.25	0.08	0.6	−0.02	0.92
Duration of hypertension	0.34	0.02	0.41	0.01	0.28	<0.05	0.23	0.17	0.12	0.34	0.23	0.12
Diagnosis of APA	0.45	<0.01	–	–	0.44	<0.01	–	–	0.58	<0.01	–	–
Log PAC	–	–	0.32	0.04			0.29	0.07	–	–	0.33	0.03

Left atrial volume index (LAVI) (ml/m^2^) = left atrial volume (ml)/BSA (m^2^), left ventricular end-diastolic diameter index (LVDdI) = left ventricular end-diastolic diameter (cm)/BSA (m^2^), left ventricular mass index (LVMI) = left ventricular mass (g)/BSA (m^2^)

## Discussion

In the present study, we reviewed echocardiographic data to investigate the difference of cardiac structure between APA and IHA. In the APA group, left atrium volume and left ventricular diameters and LVMI were greater than in IHA. Multiple linear regression analysis revealed that the diagnosis of APA and log PAC were predictors of LAVI, LVDdI, and LVMI. In the present study, we demonstrated that the morphological differences in the left atrium and left ventricle existed between the APA group and IHA group and that LVH was more progressive and common in the APA group than in the IHA group.

### Hypertension and left ventricular hypertrophy

The duration of hypertension and the number of antihypertensive agents used per patient were greater in APA than in IHA. These findings imply that hypertensive state was more severe in APA than IHA. It is well known that hypertension is one of the most important factors leading to LVH. In the present study, the duration of hypertension correlated with LVMI, although BP at the time of echocardiographic examination was similar between the two groups. These findings suggest that a more severe hypertensive status of APA in a prior period had contributed to the greater progression of LVH in APA than in IHA, although the duration of hypertension was not an independent predictor of LVMI in multiple linear regression analysis.

### Differences of cardiac structures between APA and IHA

Several lines of evidence have shown that excess aldosterone secretion promotes LVH. Brilla et al. [5, 6] demonstrated that aldosterone played an important role in excess cardiac fibrosis in a comparison of three different types of hypertensive rat models. Rossi et al. [7] demonstrated that LVH was exaggerated in patients with PA compared to patients with essential hypertension and hyperaldosteronism associated with alteration of myocardial texture suggesting increased deposition of collagen in myocardial tissue [18]. Several reports demonstrated that LVM increased more progressively in patients with PA than in patients with other types of hypertension such as essential hypertension, pheochromocytoma, and Cushing syndrome [3, 4, 7, 19]. However, there has been little information concerning the difference of left ventricular morphology among subtypes of PA. In the present study, LVMI was greater in APA than in IHA. All of the patients with APA were classified as LVH and the prevalence of LVH was higher in APA than in IHA. PAC was higher in APA and, in univariate analysis, log PAC correlated with LVMI. In addition, the multiple linear regression analysis revealed that diagnosis with APA and log PAC were independent predictors of LVMI. These findings suggest that higher aldosterone secretion in APA, at least in part, plays an important role in the greater progression of LVH in APA than in IHA through direct effects on myocardial tissue in addition to the effect secondary to hypertension. Okoshi et al. [20] demonstrated that aldosterone directory stimulates myocardial hypertrophy through the activation of protein kinase C, ERK1/2, and JNK.

Excess aldosterone secretion affects loading condition through sodium and water retention. Several studies reported that patients with APA had larger left ventricular dimensions compared to patients with other types of hypertension such as essential hypertension, pheochromocytoma, Cushing syndrome, and renovascular hypertension [4, 21, 22]. Muscholl et al. [11] reported that in patient with essential hypertension, PAC was elevated in patients with eccentric LVH compared to concentric LVH. In the present study, left atrial volume and left ventricular dimensions and diameter of the inferior vena cava were greater in APA and left ventricular wall thicknesses were similar between the two groups. These findings implied higher loading condition caused by higher aldosterone secretion in the APA group than in the IHA group. Multiple linear regression analysis revealed that LAVI significantly correlated with log PAC and LVDdI tended to be correlated with log PAC. Our findings suggested that higher aldosterone secretion stimulated sodium and water retention leading to left atrial and ventricular dilatation. Currently, to our knowledge, there is no evidence concerning the relation between the subtype of PA and left atrial volume. The change of left atrial volume would be more sensitive to change of loading condition than the left ventricle. This is the first description of the relation between the subtype of PA and left atrial volume.

In fact, in the present study, the prevalence of LVH in IHA was as high as 68 %. This high prevalence of LVH may play a role in the poor prognosis in patients with PA compared to other secondary hypertension and essential hypertension. However, it is unclear whether the difference in prognosis exists between APA and IHA. In addition, to our knowledge, there has been little information concerning the difference of left ventricular structures and function among subtypes of PA such as APA and IHA. In the present study, we demonstrated that the morphological differences in the left atrium and left ventricle existed between APA and IHA and that the degree of LVH progressed in APA compared to IHA, although it might be difficult to differentiate APA from IHA on the basis of the presence and degree of LVH. These findings may suggest the possibility of poor prognosis in patients with APA compared to IHA if they are untreated.

### Assessment of cardiac function

In the Doppler assessment of transmitral flow velocities, the value of *E* was significantly larger in APA than in IHA. This finding might indicate greater preload in APA than in IHA. The values of *A*, Dct, and *E*/*A* were similar between the two groups. In the tissue Doppler assessment of mitral annular velocities, the values of *e*′ and *E*/*e*′ in the septal and lateral sides of the mitral annulus were similar between the two groups. The values of Dct and *e*′ in both groups were below the normal range specified in the American Society of Echocardiography’s recommendation [15]. These findings suggested impairment of left ventricular relaxation in patients with PA. However, the differences of diastolic properties between APA and IHA were undetermined from our findings. Although the value of *e*′ was a good predictor of left ventricular diastolic function, it was determined by several factors such as left ventricular relaxation, preload, systolic function, and left ventricular minimal pressure [15]. Other echocardiographic parameters of diastolic properties obtained in the present study were also affected by several hemodynamic determinants. Excess aldosterone-induced higher loading condition might cause a more hyperkinetic condition in the APA group than in the IHA group and mask the difference of left ventricular diastolic properties between the two groups. In fact, in the present study, the APA group had greater left ventricular dimensions and stroke volume and similar left ventricular ejection fraction compared to the IHA group. These findings suggested that the APA group had greater cardiac output and was in a more hyperkinetic condition compared to the IHA group. Tarazi et al. [23] reported that hyperkinetic state was more frequent in patients with PA than in patients with essential hypertension because of the increase of plasma volume, an inotropic effect of aldosterone and/or enhanced sympathetic activity. However, the greater left atrial volume in APA might imply not only the difference of loading condition but also the progression of impairment of diastolic function in APA compared to IHA. In the present study, serum potassium concentration was lower in the APA group. Fitzovich et al. [24] demonstrated that chronic hypokalemia reduced left ventricular inotropic and relaxation response to epinephrine. Although lower serum potassium concentration in APA might contribute to the impairment of cardiac function, in the present study, there was no finding suggesting the progression of impairment of left ventricular systolic and diastolic function in APA compared to IHA. Loading condition caused by excess aldosterone secretion might have an effect on systolic and diastolic function as it counteracts the effects of hypokalemia.

### Study limitations

There are a few limitations in this study. This was a cross-sectional study and the number of patients was small. We could not show whether the differences of cardiac structure between APA and IHA affect their prognosis or not. In this regard, a cohort study with larger numbers of patients is needed. In our study, some patients were taking antihypertensive medications. We continued these medications owing to ethical considerations when echocardiography was performed. The influence of antihypertensive drug on our results would be small because the use of antihypertensive drugs was similar between the two groups. Despite the progression of LVH in the APA group, we could not find a difference of left ventricular diastolic properties between the two groups. Additional but more precise evaluation such as the two-dimensional speckle tracking method may be useful to evaluate the difference between the two groups.

## Conclusions

In the present study, we demonstrated that the diagnosis of APA was an independent predictor of greater left atrial volume, left ventricular dimensions, and LVM. Excess aldosterone secretion in APA compared to IHA would play a role in the progression of LVH with larger left atrial and left ventricle. The difference of left ventricular diastolic properties was unclear. To determine the usefulness of this diagnostic and prognostic parameter, a prospective study with larger numbers of subjects is needed.
